# Initial Experience With Patent Ductus Arteriosus Ligation in Pre-term Infants With Bidirectional Shunt Pattern

**DOI:** 10.3389/fped.2020.591441

**Published:** 2020-10-26

**Authors:** Ming-Chun Yang, Hsien-Kuan Liu, Hsuan-Yin Wu, Shu-Leei Tey, Yung-Ning Yang, Chien-Yi Wu, Jiunn-Ren Wu

**Affiliations:** ^1^Department of Pediatrics, E-Da Hospital, Kaohsiung City, Taiwan; ^2^School of Chinese Medicine for Post Baccalaureate, I-Shou University, Kaohsiung City, Taiwan; ^3^School of Medicine for International Students, I-Shou University, Kaohsiung City, Taiwan; ^4^Division of Cardiovascular Surgery, Department of Surgery, E-Da Hospital, Kaohsiung City, Taiwan

**Keywords:** pre-term infant, pulmonary hypertension, patent ductus arteriosus ligation, outcomes, bidirectional shunt, contraindication, surgery

## Abstract

**Background:** Patent ductus arteriosus (PDA) with a bidirectional shunt reflects critical clinical conditions. The operability of PDA with a bidirectional shunt in pre-term infants is still not clearly clarified. This study aimed to investigate the feasibility and the outcomes of PDA ligation in pre-term infants with a bidirectional shunt PDA.

**Methods:** All pre-term infants receiving PDA ligation between 2013 and 2019 were enrolled in this prospective study. Patients were allocated into two groups based on the shunting direction of PDA, which were the left-to-right group (group A) and the bidirectional group (group B). Clinical characteristics and pre-op comorbidities were analyzed. Intraoperative complications, post-op neurological sequelae, necrotizing enterocolitis, survival, and mortality were compared between these two groups.

**Results:** Thirty-seven pre-term infants were enrolled (18 in group A, 19 in group B). The mean post-menstrual age at PDA surgery was 32.0 ± 1.3 and 32.8 ± 1.5 weeks, respectively. Before surgery, 44.4 and 89.5% (group A vs. B) of the patients were using invasive mechanical ventilator (*p* < 0.01). The requirement of high-frequency oscillatory ventilatory support was significantly higher in group B. PDA rupture-related bleeding during exposing PDA or ligating PDA occurred in four infants, and all were all in group B, including one with delayed hemothorax. Early surgical mortality within 30 days of surgery was higher in group B (0 vs. 21.1%, *p* < 0.05), but only one death could be attributed to the surgery, which was caused by a pain-induced pulmonary hypertension crisis. The 5-year survival was 100% in group A, and 73.7% in group B (*p* < 0.05).

**Conclusion:** We did not recommend routine PDA ligation in pre-term infants with a bidirectional shunt. However, a bidirectional shunt should not be an absolute contraindication if they fulfill indications of PDA closure. Unexpected intraoperative PDA rupture and delayed hemothorax in a bidirectional shunt PDA should be carefully monitored. Aggressive post-op pain control is also warranted to avoid pulmonary hypertension crisis. The post-op early mortality rate was higher in the bidirectional group, which could be inherent to their poor pre-operative lung condition. Only one death was directly related to the surgery.

## Introduction

The first cry of the newborn results in lung expansion and the pulmonary pressure begins to decline soon after birth. Patent ductus arteriosus (PDA) is the most common heart disease in pre-term infants (1). Functional closure of PDA occurs within 24–48 h after birth owing to increasing PaO_2_ and decreasing circulatory vasodilators. Permanent anatomical closure occurs followed by the endothelial hyperplasia and fibrotic process (2, 3). Untreated hemodynamically significant PDA with a left-to-right shunt can result in increased pulmonary blood flow, heart failure, and pulmonary arterial (PA) hypertension (4–6), and can even progress to Eisenmenger syndrome.

Eisenmenger syndrome is an absolute contraindication for PDA closure in children and adults because the procedure could lead to pulmonary hypertension crisis, acute right ventricular failure, and even death. Pulmonary hypertension in pre-term infants is usually secondary to lung disease, mostly caused by respiratory distress syndrome and bronchopulmonary dysplasia (BPD), instead of PDA itself (7, 8). PDA with a bidirectional shunt reflects high PA pressure. Closure of hemodynamically significant PDA in pre-term infants with PA pressure lower than systemic blood pressure (left-to-right shunt) is reasonable, but PDA closure in very high PA pressure that is equal to systemic blood pressure (bidirectional shunt) is crucial because of the risk of acute right ventricular failure. However, the use of pulmonary vasodilator in pre-term infants with a bidirectional shunt will lead to a left-to-right ductal shunt and even pulmonary hemorrhage. This critical situation is more frequently encountered in extremely low-birth-weight pre-term infants. Surgical closure of hemodynamically significant PDA in pre-term infants with a bidirectional shunt remains a dilemma. Therefore, this study aimed to investigate the outcomes of hemodynamically significant PDA ligation in pre-term infants with a bidirectional shunt.

## Patients and Methods

Data of this case-control study was collected from in-patient and out-patient medical records. Ethical approval was given by the Institutional Review Board of Medical Ethics of E-DA Hospital (EMRP25106N), and all informed consents were approved by the participants' parents, with all patient information de-identified.

### Patients

**Figure 1:**
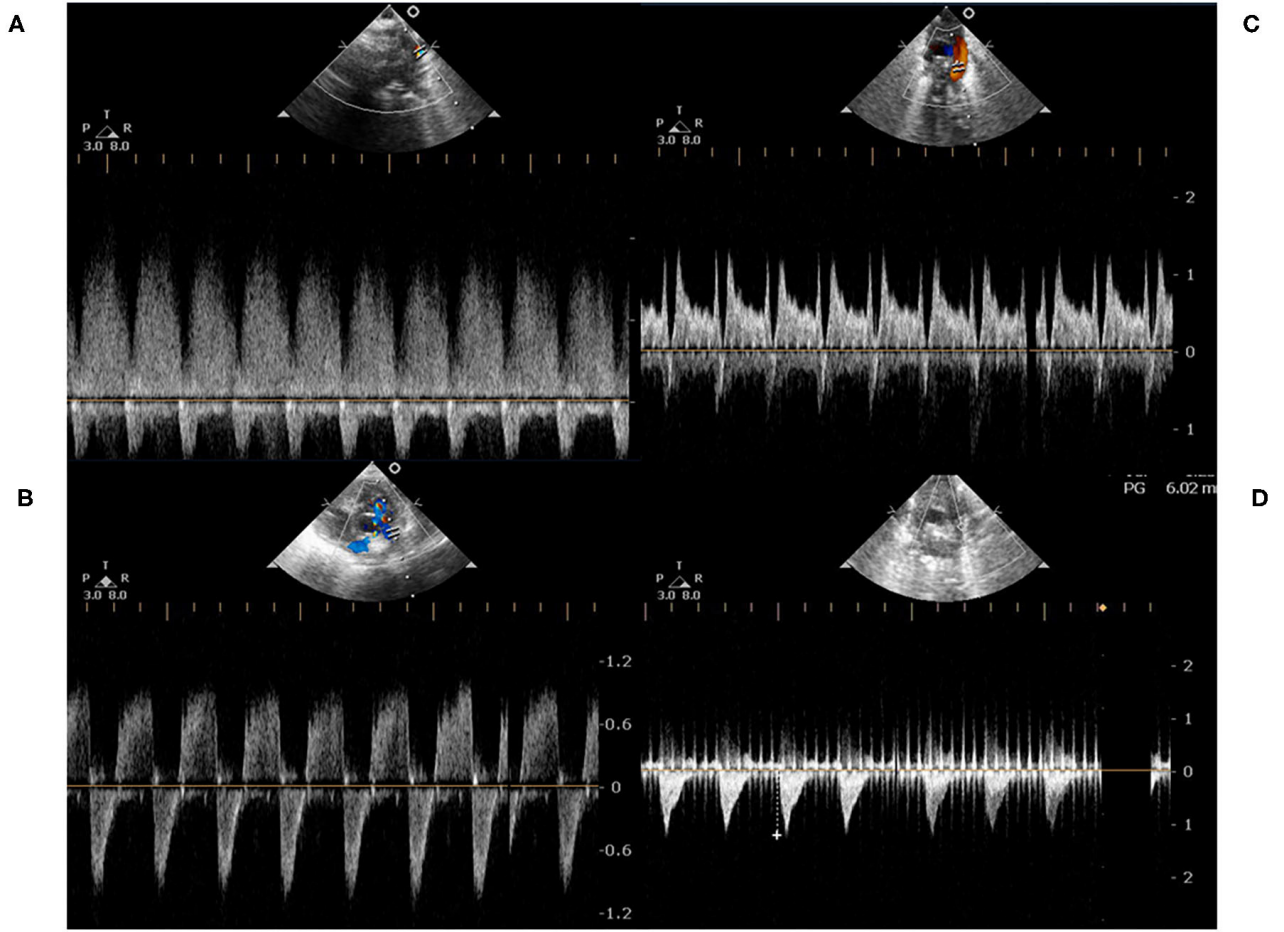
**(A)** represented a continuous left-to-right shunt of a PDA, which was detected by pulse-wave Doppler. At the end-diastolic phase, there was a persistent left-to-right shunt; **(B,C)** represented a bidirectional shunt of a PDA. At the end-diastolic phase, the shunting directions were transient right-to-left; **(D)** represented a right-to-left shunt. There was rare left-to-right shunt either at the systolic or diastolic phase.

Pre-term infants (gestational age <37 weeks) admitted to our neonatal intensive care unit undergoing PDA ligation between April 2013 and March 2019 were enrolled in our study. Patients' gender, gestational age, birth bodyweight, and delivery method were all recorded. All echocardiography was performed by the same pediatric cardiologist (MC Yang), and echocardiographic assessment was repeated on the day of surgery. PDA size, left atrium-to-aorta ratio (LA/Ao ratio), transductal velocity, and pressure gradients (between the aorta and pulmonary artery) were recorded. Patients were divided into two groups according to the shunting directions of PDA. Group A was PDA with a continuous left-to-right shunt (Figure 1A), and group B was a bidirectional shunt (Figures 1B–D). The PA pressure measured in cardiac catheterization is regarded as the gold standard for the diagnosis of pulmonary hypertension. However, it is impractical for a pre-term infant to receive a cardiac catheterization procedure only for measuring PA pressure, and this forces pediatric cardiologists to rely on echocardiographic assessment. PDA blood flow is one of the methods to evaluate PA pressure (9). The shunting directions reflect the severity of pulmonary hypertension, and PA systolic pressure was estimated by echocardiographic transductal pressure gradient assessment comparing to simultaneous systemic blood pressure. In patients with left-to-right (or right-to-left) PDA shunt at the systolic phase, the PA systolic pressure equaled systolic blood pressure subtracted (or added) by transductal pressure gradient.

In our neonatal intensive care center, the strategy of respiratory support was to use nasal continuous positive airway pressure ventilation as much as possible to lower the incidence of lung injury (10). Pre-operative and post-operative neurosonography was checked by an experienced neonatologist (HK Liu). Intraventricular hemorrhage (IVH) grading was evaluated according to a standardized assessment. Briefly, grade 1 was the bleeding area confined to the germinal matrix plus up to 10% of the ventricle volume, grade 2 was germinal matrix hemorrhage and IVH between 10 and 50% of the lateral ventricle volume, grade 3 was germinal matrix hemorrhage and IVH more than 50% of the lateral ventricle volume, and grade 4 was periventricular hemorrhagic infarction (11). Mechanical ventilatory support, necrotizing enterocolitis (NEC), and BPD were all recorded. NEC was recorded if patients presented stage 2 or 3 NEC, which was defined according to modified Bell staging (12). The diagnosis of BPD was based on oxygen or ventilator requirements, while the timing of BPD assessment was based on gestational age. Patients who were <32 weeks of gestational age were evaluated at 36 weeks post-menstrual age or when discharged home, whichever came first. Patients who were ≥32 weeks of gestational age were evaluated at 28 days of age or when discharged home, depending on which came first (13). The definition of acute kidney injury was based on Kidney Diseases: Improving Global Outcomes consensus (14). Acute kidney injury was defined if serum creatinine rose ≥0.3 mg/dL within 48 h or serum creatinine ≥1.5–1.9 times of the lowest prior serum creatinine within 7 days.

### Surgical Indications and Procedures

PDA ligations were performed by the same experienced cardiovascular surgeon (HY Wu). There were echocardiographic and clinical indications of hemodynamically significant PDA. Hemodynamically significant PDA was considered in patients fulfilling echocardiographic criteria, which were PDA diameter/bodyweight >1.3, antegrade PA diastolic flow, LA/Ao ratio ≥1.4, left ventricular enlargement, and retrograde diastolic flow in the descending aorta (15). Clinical criteria of hemodynamically significant PDA included PDA-related pulmonary hemorrhage, acute kidney injury, the need to upgrade ventilatory support, or difficulty in weaning a high-setting mechanical ventilator after excluding other etiologies. Fluid restriction to 90–100 cc/kg per day was applied if the PDA met the criteria of hemodynamically significant PDA. Once the PDA remained hemodynamically significant after fluid restriction, or fluid restriction resulted in significant electrolyte imbalance, oral ibuprofen or oral acetaminophen treatment was administered if enteral feeding had been successfully established. During the study period, intravenous ibuprofen was not commercially available in Taiwan because of small marketing requirements, which limited our use of ibuprofen in candidates. Surgical indications of PDA ligation were hemodynamically significant PDA plus one of the following (1) pre-term infants who could not tolerate enteral trophic feeding so that oral ibuprofen/acetaminophen could not be given; (2) infants who failed to respond to oral ibuprofen/acetaminophen treatment; (3) infants who were contraindicated to receive oral ibuprofen (active pulmonary hemorrhage, severe IVH, thrombocytopenia <100,000/uL, NEC, urine output <0.5 ml/kg/h, serum creatinine >1.7 mg/dL).

Surgical PDA ligation was performed via left posterolateral thoracotomy in the intensive care unit. The incision was carried out in the left third or fourth intercostal space at the right decubitus position. The ductus and the recurrent laryngeal nerve were exposed with the aid of a dissector. After PDA was exposed, a ligature was passed around PDA, gently tied town, and then a metal clip was placed for additional security. Body temperature, heart rate, and blood pressure were closely monitored and recorded right before the surgery and within 24 h after PDA ligation. Continuous intravenous morphine infusion (0.01 mg/kg/h) was given as a pain control protocol until 48 h after surgery.

### Primary Outcome

Primary outcomes referred to complications occurring at or soon after surgery, including PDA rupture-related bleeding during the surgery, desaturation or unstable saturation during the surgical procedure that required temporary interruption of surgery, post-op pneumothorax, post-op hemothorax, vocal cord paralysis, phrenic nerve palsy, and immediate surgical mortality on the day of surgery.

### Secondary Outcome

Secondary outcomes were complications within 30 days of the surgery, including neurological sequelae, NEC, and early mortality (16). BPD severity and 5-year survival were also included in secondary outcomes.

IVH progression was defined in patients whose IVH grading increment was equal to or more than 2 grades. Progression to periventricular leukomalacia (PVL) was defined in patients who had no PVL before PDA ligation, yet evolved into PVL after surgery at follow-up. Because early mortality might deter infants from developing post-op PVL, post-op NEC or BPD, these secondary outcomes were also adjusted into composite outcomes, including “early mortality or progression to PVL,” “early mortality or post-op NEC,” and “early mortality or BPD” to avoid comorbidity bias caused by early mortality.

## Statistical Analysis

Data were analyzed by SPSS version 22 statistical software. Continuous variables were presented as mean ± standard deviation (SD). Independent Student's *t*-test was applied for comparison of mean values of the two groups, and the Mann-Whitney *U*-test was used to compared means if the data were not normally distributed. Pearson's chi-square test and Fisher's exact test were used to compare categorical variables, while the Kaplan-Meier model was applied to analyze survival, and the log-rank test was used to compare survival between the two groups. Statistical significance was defined as a *p*-value < 0.05.

## Results

Thirty-seven patients were enrolled in this study, including 18 in group A, and 19 in group B. The demographic characteristics and pre-op conditions are shown in Table 1. The mean age at surgery was 20.4 and 13.2 days old (3.1–37.7 vs. 1.7–24.9, 95% confidence interval). Respiratory distress was more severe in group B. In group A, nine infants had failure of non-invasive ventilatory support (including failure of nasal continuous positive airway pressure support in six infants, and failure of nasal intermittent positive airway pressure support in three infants) caused by hemodynamically significant PDA. The need to upgrade the setting of the original invasive mechanical ventilator was 50% (9/18) in group A, and 89.5% (17/19) in group B. One out of 18 infants (5.6%) in group A required high-frequency oscillatory ventilator, whereas 8 of 19 infants (42.1%) in group B required high frequency oscillatory ventilatory support (*p* < 0.05). Of those under invasive ventilatory support, the oxygen index was significantly higher in group B (4.7 ± 2.2 vs. 9.3 ± 2.7, *p* < 0.05). Two infants (2/37) in group B used inhaled nitric oxide to maintain adequate oxygenation, and one (1/37) of them also took sildenafil.

**Table 1:** Demographic characteristics and pre-operative conditions.

	**L-to-R shunt**	**Bidirectional shunt**	***P*-value**
Number	18	19	
Gestational age (week-old)	29.1 ± 0.8	30.9 ± 1.2	0.22
Birth body weight (gram)	1329 ± 201	1452 ± 187	0.66
Vaginal delivery	10 (55.6%)	5 (26.3%)	0.09
Male	9 (50%)	11 (57.9%)	0.63
Antenatal corticosteroid	7 (38.9%)	3 (15.8%)	0.11
Age at surgery (day-old)	20.4 ± 8.2	13.2 ± 5.5	0.22
Age at surgery (PMA, week-old)	32.0 ± 1.3	32.8 ± 1.5	0.68
Body weight at surgery (gram)	1544 ± 264	1585 ± 216	0.90
**Pre-op complications**			
Severe IVH	2 (11.1%)	4 (21.1%)	0.66
Periventricular leukomalacia	1 (5.6%)	1 (5.3%)	1.00
Meconium ileus	1 (5.6%)	0 (0%)	0.47
NEC	1 (5.6%)	0 (0%)	0.47
Pulmonary hemorrhage	5 (27.8%)	9 (47.4%)	0.31
Acute kidney injury	10 (55.6%)	11 (57.9%)	0.67
**Pre-op ventilatory support**			
Non-invasive ventilator or no support	9 (50%)	2 (10.5%)	<0.01*
Invasive ventilator	9 (50%)	17 (89.5%)	<0.01*
Conventional ventilator	8 (44.4%)	9 (47.4%)	0.09
HFOV	1 (5.6%)	8 (42.1%)	0.012*
Inhaled nitric oxide	0	2 (10.5%)	
Oxygen index	4.7 ± 2.2	9.3 ± 2.7	0.02*
LA/Ao ratio	1.73 ± 0.07	1.73 ± 0.08	0.97
PDA size/BW (mm/kg)	2.1 ± 0.2	2.4 ± 0.2	0.33
Cardiothoracic ratio on radiograph	0.60 ± 0.05	0.65 ± 0.08	0.74
Serum creatinine before PDA closure (mg/dL)	1.1 ± 0.6	1.4 ± 1.0	0.21
Prior ibuprofen/acetaminophen treatment	7 (38.9%)	3 (15.8%)	0.11

HFOV, high frequency oscillatory ventilator; IVH, grade 3 or 4 intraventricular hemorrhage; LA/Ao ratio, left atrium-to-aorta ratio; NEC, stage 2 or 3 necrotizing enterocolitis; PMA, post-menstrual age; * represented statistically significant, p < 0.05.

The LA/Ao ratio, PDA size indexed by body weight at surgery (PDA size/BW), and the cardiothoracic ratios on chest radiograph were similar between the two groups. There was no significant difference in serum creatinine level or incidence of acute kidney injury before surgery. The use of oral ibuprofen treatment was limited because seven patients developed acute kidney injury with serum creatinine >1.7 mg/dL, 17 patients were restricted from enteral feeding due to shock status requiring dopamine infusion, 14 patients developed active pulmonary hemorrhage, and one patient had NEC. Only ten infants received oral ibuprofen/acetaminophen before surgery.

### Primary Outcomes

Perioperative vital signs are shown in Table 2. Infants in group B had a higher incidence of shock requiring dopamine infusion prior to the surgery (*p* < 0.05). However, under continuous dopamine infusion before surgical ligation, the systolic, diastolic, and mean blood pressure levels were similar between the two groups. Group B had higher estimated PA systolic pressure compared to group A (58 ± 3 vs. 41 ± 2 mmHg, *p* < 0.001) on the day of surgery. Milrinone, as a medical treatment of PDA in whom had congestive heart failure and reduced left ventricular contractility, was administered in five infants before surgery. After PDA ligation, there was no infant experiencing post-ligation syndrome, so adding of inotropes was not required.

**Table 2:** Perioperative vital signs of the patients receiving PDA ligation.

	**L-to-R shunt *n* = 18**	**Bidirectional shunt*n* = 19**	***P*-value**
Pre-op body temperature (°C)	36.7 ± 0.1	36.4 ± 0.2	0.23
Pre-op heart rate (bpm)	162 ± 2	165 ± 3	0.58
Tachycardia > 160 bpm	12	13	0.91
Use of dopamine	5	12	0.03^*^
**Pre-op blood pressure (mmHg)**			
Systolic blood pressure	64 ± 3	63 ± 3	0.91
Diastolic blood pressure	36 ± 2	35 ± 3	0.80
Mean blood pressure	45 ± 2	42 ± 2	0.83
Estimated PASP (mmHg)	41 ± 2	58 ± 3	<0.001^*^
Post-op body temperature (°C)	36.6 ± 0.1	36.6 ± 0.1	0.74
Post-op heart rate (bpm)	165 ± 3	166 ± 4	0.75
**Post-op blood pressure (mmHg)**			
Systolic blood pressure	66 ± 4	67 ± 5	0.88
Diastolic blood pressure	42 ± 3	44 ± 4	1.00
Mean blood pressure	50 ± 3	50 ± 4	0.95

Bpm, beats per minute; PASP, pulmonary arterial systolic pressure;

* *represented statistically significant, p < 0.05*.

The overall intraoperative and immediate post-operative complications are listed in Table 3. These complications included PDA rupture-related PDA bleeding (10.8%), intra-op desaturation (10.8%), post-op pneumothorax (8.1%), delayed post-op hemothorax (2.7%), vocal cord paralysis (2.7%), and phrenic nerve palsy (5.4%).

**Table 3:** Primary outcome–immediate complications after PDA ligation.

	**L-to-R shunt *n* = 18**	**Bidirectional shunt*n* = 19**	***P*-value**
PDA bleeding during surgery	0 (0%)	4 (21.1%)	0.04^*^
Intraoperative desaturation	1 (5.9%)	3 (15.8%)	0.32
Pneumothorax	1 (5.9%)	2 (10.5%)	0.62
Hemothorax	0 (0%)	1 (5.3%)	0.34
Vocal cord paralysis	1 (5.9%)	0 (0%)	0.28
Phrenic nerve palsy	2 (11.8%)	0 (0%)	0.12
Immediate surgical mortality	0 (0%)	0 (0%)	NS

NS, non-significant;

* *represented statistically significant, p < 0.05*.

PDA rupture-related bleeding during exposing PDA or ligating PDA occurred in four infants in group B, whereas no infant experienced PDA rupture in group A (*p* < 0.05). Intra-op desaturation requiring temporary interruption of surgery occurred in four infants (1 in group A, and 3 in group B). Although it was non-significantly different, intra-op desaturation occurred more frequently in group B. One infant in group B received emergent chest tube insertion on the day of surgery because of delayed hemothorax, which was considered to be caused by PDA rupture-related bleeding. Her condition stabilized soon after the intervention. There was no immediate surgical mortality on the day of surgery in both groups.

### Secondary Outcomes

**Figure 2:**
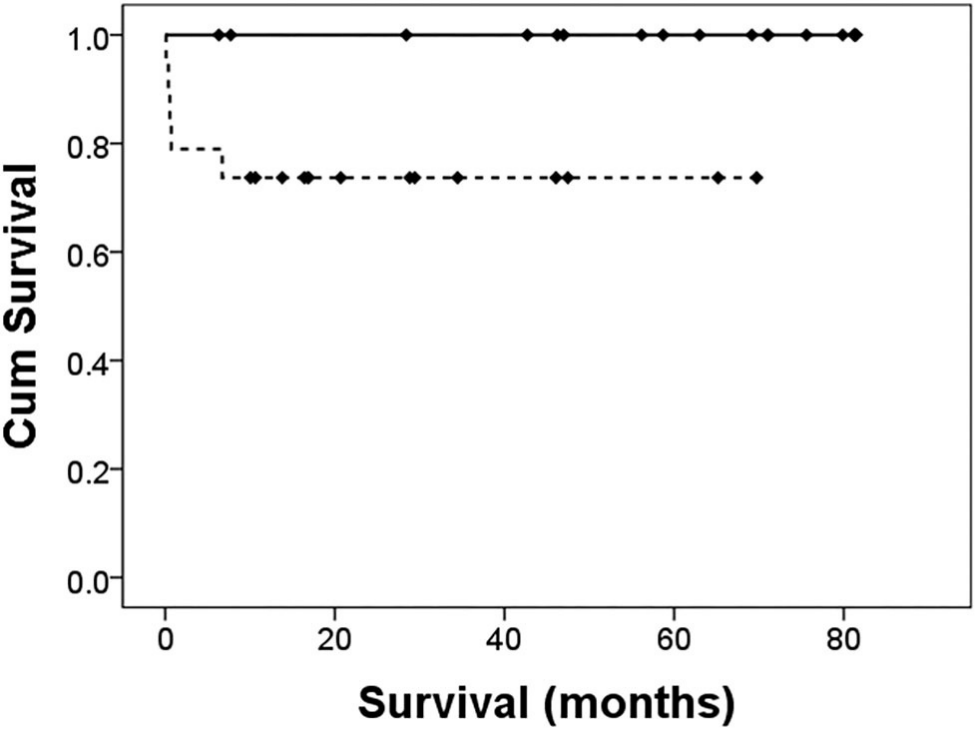
Cumulative survival after PDA ligation. The solid line is the left-to-right group. The dotted line is the bidirectional group. The Log-rank test revealed a significant difference in survival, *p* = 0.02.

As shown in Table 4, after PDA ligation, there were more infants in group A developing progression to PVL (33.3% in group A vs. 5.3% in group B, *p* < 0.05). Two infants developed NEC after surgery, and both of them were in group B. The overall incidence of BPD was 88.9% in group A, and 47.4% in group B (*p* = 0.054). Of the different severity of BPD, the incidence was similar between the two groups. Early mortality occurred only in group B (four infants, 21.1%). The causes of death were sepsis in two infants, stage 3 NEC in one infant, and pulmonary hypertensive crisis in one infant, with this infant expiring on the next day of surgery. Among the composite outcomes, only “early mortality or NEC” was significantly different (0% in group A vs. 26.3% in group B, *p* < 0.05). Although the overall mortality was higher in group B than that in group A (26.3 vs. 0%, *p* < 0.05), only one death could be attributed to PDA ligation. As shown in Figure 2, the 5-year survival was higher in group A (100 vs. 73.7%, *p* = 0.02).

**Table 4:** Secondary outcomes–late adverse outcomes after PDA ligation.

	**L to R shunt *n* = 18**	**Bidirectional shunt*n* = 19**	***P*-value**
Neurological complications			
Post-op severe IVH	6 (33.3%)	5 (26.3%)	0.72
IVH progression	4 (22.2%)	1 (5.3%)	0.15
Post-op PVL	6 (33.3%)	4 (21.1%)	0.46
Progression to PVL	6 (33.3%)	1 (5.3%)	0.04^*^
Hydrocephalus	2 (11.1%)	3 (15.8%)	0.63
Post-op NEC	0	2 (10.5%)	0.16
Bronchopulmonary dysplasia			
Overall	16 (88.9%)	9 (47.4%)	0.054
Mild	3 (16.7%)	0	0.10
Moderate	1 (5.6%)	0	0.36
Severe	12 (66.6%)	9 (47.4%)	0.71
Early mortality	0	4 (21.1%)	0.04^*^
Overall Mortality	0	5 (26.3%)	0.02^*^
Composite outcomes			
Early mortality or progression to PVL	6 (33.3%)	7 (36.8%)	0.82
Early mortality or post-op NEC	0	5 (26.3%)	0.02^*^
Early mortality or BPD	16 (88.9%)	13 (68.4%)	0.13
Death after PDA ligation (days)	NA	29 ± 19	NA

BPD, Bronchopulmonary dysplasia; IVH, intraventricular hemorrhage; NEC, Necrotizing enterocolitis; PVL, Periventricular leukomalacia; NA, not applicable;

* *represented statistically significant, p < 0.05*.

## Discussion

This is a novel study of PDA closure in pre-term infants exploring the relationship between the shunting directions of PDA and surgical outcomes in the literature. There are several unique important findings in this study. Pre-term infants with a bidirectional shunt PDA were prone to have higher PA pressures, unstable blood pressures, and inherent worse respiratory conditions requiring more invasive mechanical ventilatory support. PDA rupture-related bleeding during the surgical procedure was a major challenge for both medical staff and patients, especially while exposing or ligating the PDA. Although there was no significant difference, intra-op desaturation occurred more frequently in the bidirectional group. Unexpected vascular bleeding and unstable oxygen saturation complicated the surgical procedure. Even after the procedure, delayed hemothorax several hours after PDA ligation still occurred and required emergent chest tube insertion in one infant. Among the composite outcomes of early mortality and post-op comorbidities, only NEC was more frequent in group B. NEC was a major concern that complicated clinical care after PDA surgery in group B. In most patients with bidirectional shunt pattern, PDA ligation was feasible. Only one death died of pulmonary hypertensive crisis could be attributed to PDA surgery. However, early mortality of 21.1% was observed.

The operability of PDA in PA pressure reaching systemic blood pressure is generally not feasible in children and adults. Eisenmenger syndrome remains an absolute contraindication of PDA closure in children and adults (17). The major concerns are post-op pulmonary hypertensive crisis and acute right heart failure because of irreversible pulmonary arterial hypertension (18–23). Nevertheless, pulmonary arterial smooth muscle cell hypertrophy and endothelial cell proliferation are believed to be the main pathophysiology in irreversible pulmonary hypertension, but it rarely happens before 1 year of age (24, 25).

Early PDA treatment within 7 days of birth is generally not recommended because of possible spontaneous closure in the first week, and early treatment is associated with higher mortality and higher BPD rate (26). In this cohort, six infants (two in group A, four in group B) underwent PDA ligation within 7 days old. Among these six infants, four infants had pulmonary hemorrhage, one infant developed PDA-related shock, and one infant had both pulmonary hemorrhage and shock. Emergent surgical ligation was performed in three infants because of refractory shock and persistent pulmonary hemorrhage. Another three infants with pulmonary hemorrhage also underwent surgical ligation after the failure of controlling PDA with fluid restriction.

In our study, most of the infants with bidirectional shunt were mainly left-to-right shunt (Figures 1B,C), but two infants were bidirectional with a mainly right-to-left shunt (Figure 1D). In pre-term infants, the PA pressures were dynamic and influenced by the lung condition. The persistent left-to-right shunt PDA (Figure 1A) or bidirectional pattern with mainly left-to-right shunt PDA (Figures 1B,C) ultimately contributed to pulmonary blood overflow and pulmonary edema. The pulmonary blood overflow further led to pulmonary hemorrhage at an unexpected timing. The occurrence would be abrupt within hours, especially in extremely low birth weight infants. Once pulmonary hemorrhage occurred, the PA pressure would suddenly elevate to the systemic level, which in turn caused right-to-left shunt. Medical management of pulmonary hemorrhage eventually stabilized the edema or hemorrhage, and the PA pressure would return to a lower level. However, the PDA became left-to-right shunt again afterward. This entered an endless vicious cycle, so that interruption of the cycle by ligating PDA provided benefit to these pre-term infants because no one could predict when would be the next episode of pulmonary hemorrhage. This was also the rationale why we needed to perform PDA ligation in an infant with a bidirectional shunt.

Because of the preserved reversibility of pulmonary hypertension, PDA ligation was performed in two particular pre-term infants with a mainly right-to-left shunt. Both of these infants were treated with high-frequency oscillatory ventilator, and were also the only two infants receiving inhaled nitric oxide during PDA ligation. One of these, whose birth body weight was 845 gram, had grade 4 RDS and severe persistent pulmonary hypertension of the newborn that was refractory to intratracheal surfactant administration, experiencing repeated pulmonary hemorrhage due to a transient left-to-right shunt and pulmonary overflow while inhaling 10 ppm nitric oxide. Pulmonary hemorrhage further aggravated pulmonary hypertension and led to the reappearance of a right-to-left shunt. Emergent PDA ligation was performed to interrupt the endless vicious cycle in this infant at the age of 2 days. He was weaned off the invasive mechanical ventilator successfully under intensive respiratory care and other medical treatments when he was 13 days old, and was discharged at corrected age of 52 days.

The other infant, whose birth bodyweight was 500 grams, was born at gestational age 27 weeks. He also received PDA ligation in the status of a right-to-left shunt. In the beginning, PDA was not hemodynamically significant, so it was treated conservatively. His pulmonary condition had never been well, so a high ventilator setting with intermittent administration of inhaled nitric oxide had been applied for 3 months. Weaning of invasive ventilator was not achievable in the first 3 months of life. Echocardiography at corrected age of 6 days revealed a 2.5 mm right-to-left shunt PDA. After titrating up nitric oxide, PDA became a bidirectional shunt on echocardiography. This response indicated the reversibility of pulmonary vasoreactivity. Because high mechanical ventilator setting and inhaled nitric oxide were unable to be weaned off successfully in the past 3 months, we decided to carry out PDA ligation and then use inhaled nitric oxide continuously to treat his severe pulmonary hypertension. PDA ligation was performed at the corrected age of 8 days. The PDA was edematous and fragile. PDA rupture happened intraoperatively, otherwise the whole surgical procedure was smooth. Afterwards, the patient was successfully weaned from an invasive mechanical ventilator 37 days after the operation, by the time he was at the corrected age of 44 days. Inhaled nitric oxide was shifted to oral sildenafil for the treatment of pulmonary hypertension. Although this patient had severe BPD, he needed oxygen hood with low oxygen fraction only. Our limited experience demonstrates the feasibility of PDA closure in pre-term infants with severe pulmonary hypertension, even though PA pressure was higher than systemic blood pressure.

There was an infant in group B who died from a pulmonary hypertensive crisis on the next day of PDA ligation. This infant's condition was stable initially after surgery, but he developed circulatory shock and desaturation 6 h later. Despite inhaled nitric oxide being administered, the patient still had acute right ventricular failure and shock. In this case, routine morphine was administered continuously after surgery, but the dosage was not increased while he had the severe pulmonary hypertensive crisis. On reflection, the pain-induced pulmonary hypertensive crisis was a major problem leading to death, and from this case, it was determined that management of pain control should be more aggressive in infants with very high PA pressure after PDA surgery.

There was a higher incidence of intra-operative PDA bleeding and post-op hemothorax in group B. Two of the four infants in group B with PDA bleeding received inhaled nitric oxide during surgery. The postulated hypothesis was that the intraductal blood pressure was very high because the PA pressure was approaching systemic blood pressure, which led to engorgement and fragility of the PDA. Once touching the vessel while exposing or ligating the PDA, a rupture occurred easily and resulted in bleeding. Inhaled nitric oxide also contributed to the bleeding because the exposure of nitric oxide might lead to a certain degree of coagulopathy (27). Transcatheter PDA closure in pre-term infants has become an available intervention and several trials presented promising outcomes (28, 29). The hemodynamic study can be measured during the cardiac catheterization, which even allows a test occlusion before PDA closure in pre-term infants with a bidirectional shunt. Our study demonstrated the feasibility of PDA ligation in pre-term infants with a bidirectional shunt. Although intra-operative PDA bleeding and post-op hemothorax was a concern in surgery, transcatheter closure might be an alternative choice to overcome the bleeding-related complications.

## Limitations

This was a prospective case-control study, but the allocation of patients and the timing of surgery were not randomized. We allocated these patients into groups A or B according to their echocardiographic PDA blood flow findings on the day of surgery. The pre-op respiratory condition was worse in group B, and this might have influenced the post-operative outcomes. Due to the small marketing requirement of intravenous ibuprofen in Taiwan, the intravenous form had not been available until 2019. This resulted in few candidates receiving intravenous ibuprofen in our cohort. Hence, the experience of ibuprofen treatment may be quite different from other countries. Owing to the background, we had more infants undergoing surgical ligation, even in the status of a bidirectional shunt.

Approximately one-fourth of infants with moderate to severe BPD developed pulmonary hypertension later in their life (30). In our cohort, 22 infants developed moderate to severe BPD (13 in group A, nine in group B). Although group B had higher estimated PA pressure during the surgery, the development of BPD-related pulmonary hypertension was not evaluated.

There were only 37 pre-term infants enrolled in this study, so statistical calculations might not have reached significance because of the relatively small number of patients. No studies have focused on outcomes of PDA with a bidirectional shunt in pre-term infants. This can be regarded as an initial experience for similar subjects, and the conclusions would be more confidently regarded as valid if more patients could be enrolled in future research.

## Conclusions

This is a novel study exploring outcomes of PDA ligation in pre-term infants with a bidirectional shunt. Post-op echocardiography revealed favorable left ventricular and right ventricular systolic function and no signs of acute right ventricular failure in our study, except for one patient expiring from a pulmonary hypertensive crisis. The relatively high mortality rate in group B could be attributed to poor pre-op conditions, including higher oxygen index and unstable blood pressure requiring dopamine infusion. Post-ligation outcomes proved the feasibility of PDA closure in critical pre-term infants with severe pulmonary hypertension and even with a bidirectional shunt. Composite outcomes showed neurological sequelae and the incidence of BPD did not differ between the two groups. According to this result, we did not recommend routine PDA ligation in pre-term infants with a bidirectional shunt. However, a bidirectional shunt should not be an absolute contraindication if pre-term infants fulfill indications of PDA closure. Surgeons need to be extremely careful while dissecting the tissue, exposing and ligating the PDA. Even though surgical ligation might be successfully performed, watchful monitoring for delayed hemothorax is still warranted. Post-operative pain control should be very aggressive if there are any signs of a pulmonary hypertension crisis.

## Data Availability Statement

All datasets generated for this study are included in the article/supplementary material.

## Ethics Statement

The studies involving human participants were reviewed and approved by Institutional Review Board of Medical Ethics of E-DA Hospital. Written informed consent to participate in this study was provided by the participants' legal guardian/next of kin.

## Author Contributions

The ideas and protocol of this study were designed by M-CY. Medical records were obtained by M-CY, H-KL, H-YW, S-LT, Y-NY, and C-YW. M-CY and H-KL completed the manuscript and worked for the statistical analysis. J-RW revised the article. All authors contributed to the article and approved the submitted version.

## Conflict of Interest

The authors declare that the research was conducted in the absence of any commercial or financial relationships that could be construed as a potential conflict of interest.

## Abbreviations

LA/Ao ratio: left atrium-to-aorta ratio
HFOV: high frequency oscillatory ventilator
PA: pulmonary arterial
PASP: pulmonary arterial systolic pressure
PMA: post-menstrual age
PVL: periventricular leukomalacia.
